# SARS-CoV-2 Spike- and Nucleoprotein-Specific Antibodies Induced After Vaccination or Infection Promote Classical Complement Activation

**DOI:** 10.3389/fimmu.2022.838780

**Published:** 2022-07-04

**Authors:** Rachel E. Lamerton, Edith Marcial-Juarez, Sian E. Faustini, Marisol Perez-Toledo, Margaret Goodall, Siân E. Jossi, Maddy L. Newby, Iain Chapple, Thomas Dietrich, Tonny Veenith, Adrian M. Shields, Lorraine Harper, Ian R. Henderson, Julie Rayes, David C. Wraith, Steve P. Watson, Max Crispin, Mark T. Drayson, Alex G. Richter, Adam F. Cunningham

**Affiliations:** ^1^ Institute of Immunology and Immunotherapy, University of Birmingham, Birmingham, United Kingdom; ^2^ Institute of Cardiovascular Sciences, University of Birmingham, Birmingham, United Kingdom; ^3^ School of Biological Sciences, University of Southampton, Southampton, United Kingdom; ^4^ Periodontal Research Group, School of Dentistry, Institute of Clinical Sciences, University of Birmingham, and Birmingham Community Healthcare National Health Service Trust, Birmingham, United Kingdom; ^5^ Department of Critical Care Medicine, University Hospitals Birmingham National Health Service (NHS) Trust, Birmingham, United Kingdom; ^6^ Institute of Applied Health Research, University of Birmingham, Birmingham, United Kingdom; ^7^ Institute for Molecular Bioscience, University of Queensland, St Lucia, QLD, Australia

**Keywords:** complement, COVID-19, SARS-CoV-2, vaccine, antibodies

## Abstract

Antibodies specific for the spike glycoprotein (S) and nucleocapsid (N) SARS-CoV-2 proteins are typically present during severe COVID-19, and induced to S after vaccination. The binding of viral antigens by antibody can initiate the classical complement pathway. Since complement could play pathological or protective roles at distinct times during SARS-CoV-2 infection we determined levels of antibody-dependent complement activation along the complement cascade. Here, we used an ELISA assay to assess complement protein binding (C1q) and the deposition of C4b, C3b, and C5b to S and N antigens in the presence of antibodies to SARS-CoV-2 from different test groups: non-infected, single and double vaccinees, non-hospitalised convalescent (NHC) COVID-19 patients and convalescent hospitalised (ITU-CONV) COVID-19 patients. C1q binding correlates strongly with antibody responses, especially IgG1 levels. However, detection of downstream complement components, C4b, C3b and C5b shows some variability associated with the subject group from whom the sera were obtained. In the ITU-CONV, detection of C3b-C5b to S was observed consistently, but this was not the case in the NHC group. This is in contrast to responses to N, where median levels of complement deposition did not differ between the NHC and ITU-CONV groups. Moreover, for S but not N, downstream complement components were only detected in sera with higher IgG1 levels. Therefore, the classical pathway is activated by antibodies to multiple SARS-CoV-2 antigens, but the downstream effects of this activation may differ depending the disease status of the subject and on the specific antigen targeted.

## Introduction

Infection with SARS-CoV-2, the causative agent of COVID-19, results in a spectrum of clinical presentations ranging from asymptomatic infections to severe disease and death. Although some factors that can predict risk of severe disease are known, such as obesity or age, it is clear that other host factors, including immune status, also contribute (1–3). Thus, it is likely that COVID-19 represents a collection of syndromes, caused by one pathogen, where disease severity is influenced by host and pathogen factors.

Two antigens that are common targets of the immune response to SARS-CoV-2 are the spike (S) glycoprotein, which is essential for both binding and entry into host cells, and the nucleocapsid (N) protein, involved in packaging the genomic material (4). Antibodies to these antigens are induced after infection, and antibodies to S glycoprotein can be protective (5–7). Indeed, the S glycoprotein is the sole SARS-CoV-2 viral antigen targeted by all current licensed vaccines (8). After natural infection of non-vaccinated individuals, although the appearance of antibodies to both of these antigens can occur early in mild disease, the presence of such antibodies is usually well-established at times severe disease develops. This means that substantial levels of viral antigen may still be present within the host for these antibodies to bind (9). In contrast, in most vaccinated individuals who have not previously been infected, high levels of antibodies to S are present when they subsequently encounter the pathogen. This means that there can be circumstances when: i) there are concomitant high-levels of antibodies to S and N as well as relatively high-levels of antigen (antibodies induced during infection) and ii) high-levels of antibodies to S and relatively low-levels of antigen (infection of vaccinated, previously naïve individuals).

After antigen binding by antibody, complement activation can occur through the classical pathway (10). This cascade requires C1q binding to antibody and the generation of a C3 convertase derived in part from C4, through the production of C4b. This results in the cleavage of C3 and C5, with C3b and C5b forming a complex proximal to the site of antibody binding. The activation of the complement cascade may have positive or negative effects for the host associated with the timing of its activation and possibly the different pathways involved (11–15).

To improve our understanding of the relationship between SARS-CoV-2-specific antibodies and complement activation, we developed a solid phase C1q-binding assay and C4b, C3b and C5b complement deposition assays using S and N proteins from the SARS-CoV-2 Wuhan strain. These studies identified differences in antibody-associated complement activation that were associated with the stage of infection in the host.

## Methods

### Ethics and Patient Samples

Sera were obtained from distinct groups of subjects from well-validated cohorts that are described below (3, 5). Group 1: Non-vaccinated individuals without any reported COVID infection (NEG). Sera were obtained from subjects in May 2020, prior to widespread PCR testing and before the introduction of vaccines against SARS-CoV-2 infection. Sera were screened using a clinically validated, CE marked, ELISA assay that measures the IgG, IgA and IgM (IgGAM) response to the S glycoprotein (16, 17) (manufactured by The Binding Site (TBS; product code: MK654), Birmingham). This assay, described below in the section on detecting antibodies to S and N, has been clinically validated and reported to have a sensitivity of 98.6% (95% CI, 92.6-100.0%) and a specificity of 98.3% (95% CI, 96.4-99.4%) (16). Group 2: Individuals without evidence of infection (as determined by an absence of anti-N antibodies), vaccinated 28-35 days previously with BNT162b2 vaccine (VACC). Group 3: Individuals without evidence of infection (as determined by an absence of anti-N antibodies) who had received their second dose of BNT162b2 vaccine at least 28 days previously (DOUBLE VACC). Group 4: These sera were obtained in May 2020 from a cohort of healthcare workers from the University Hospitals Birmingham Foundation Trust, who had previously self-isolated a minimum of 28 days previously because they experienced symptoms suggestive of COVID-19, and had not been hospitalized for any of these symptoms. In May 2020 widespread PCR testing was not available, and thus most of these samples were not from individuals with prior confirmed PCR tests. Since the only predefined exclusion criteria was participation in existing SARS-CoV-2 vaccine trial or current COVID-19 symptomatology and the time was prior to the introduction of vaccines, anti-S antibodies could be used as a reliable surrogate of previous infection. Anti-S IgGAM were determined using the clinically validated anti-S glycoprotein ELISA described above (non-hospitalised convalescents, NHC). Group 5: Non-vaccinated convalescent, PCR confirmed SARS-CoV-2 infection patients who required ITU treatment, samples taken a minimum of 4 months after ITU discharge (ITU-CONV).

Ethical approval for obtaining samples for groups 1 -4 was provided by the London – Camden and Kings Cross Research Ethics Committee reference 20/HRA/1817. Ethical approval for obtaining samples for group 5 was provided by the North West ethics committee, Preston CIA UPH IRAS approval reference REC 20\NW\0240.

### Antigens Used in This Study

To generate the spike glycoprotein used in this study, HEK293F cells were transiently transfected with a pα-H plasmid containing the near full-length sequence for the Wuhan SARS-CoV-2 spike (GenBank: MN908947). The spike glycoprotein used here contains 1208 amino acids and includes all the S1 and most of the S2 domain (18, 19). The protein has been modified so that there are an additional four prolines present in addition to the two which are normally expressed (2P) to stabilize recombinant spike (18). The 6 prolines are present at positions 817, 892, 899, 942, 986 and 987 (18). This so-called HexaPro spike glycoprotein expresses as a metastable recombinant SARS-CoV-2 prefusion ectodomain. Extensive comparisons between the native, 2P and HexaPro spike glycoproteins demonstrate that they have comparable native-like protein architecture, have similar antigenic properties including the induction of neutralizing antibodies (20–22) and have similar glycosylation profiles (18, 19, 23, 24).

The HEK293F cells were cultured in Freestyle 293 Expression medium (Fisher Scientific) and maintained at a density of 0.2 x 10^6^ cells/mL at 37°C, 8% CO_2_ and 125 rpm shaking. Prior to transfection, two solutions of 25 mL Opti-MEM (Fisher Scientific) medium were prepared. The expression plasmid encoding SARS-CoV-2 HexaPro was added to the first solution to give a final concentration of 310 µg/L. To the other solution, 1 mg/mL pH7 polyethylenimine (PEI) max reagent was added to generate a ratio of 3:1 PEI max:plasmid DNA. Both solutions were combined and incubated at room temperature for 30 minutes. Cells were transfected at a density of 1 x 10^6^ cells/mL and incubated for 7 days at 37°C, 8% CO_2_ and 125 rpm shaking.

Cells were centrifuged at 3041g for 30 minutes at 4°C and supernatant was applied to a 500 mL Stericup-HV sterile vacuum filtration system (Merck) with a pore size of 0.22 µm. Purification of HexaPro S protein was undertaken using an ÄKTA Pure system (Cytiva). A 5 mL HisTrap Excel column (Cytiva) charged with Ni(II) was equilibrated using 10 column volumes (CV) of wash buffer (50 mM Na_2_PO_4_, 300 mM NaCl, pH 7). Supernatant was then loaded onto the column at a flow rate of 5 mL/min and washed with 10 CV of washing buffer containing 50 mM imidazole. Protein was eluted from the column in 3 CV of elution buffer (300 mM imidazole in washing buffer) and buffer exchanged to phosphate buffered saline (PBS) and concentrated using a Vivaspin column (MWCO 100 kDa) (Cytiva).

The nickel purified eluate was concentrated to 1 mL in PBS and injected into a Superdex 200 pg 16/600 column (Cytiva) to further purify trimeric S protein using size exclusion chromatography (SEC). The column was washed with PBS at 1 mL/min for 2 hours where fractions corresponding to the correct peak on the size exclusion chromatogram were collected and concentrated to ~1 mL as above.

Nucleocapsid was generated as a recombinant protein from *E. coli* by the Protein Expression Facility at the University of Birmingham (17).

### Detection of Antibodies Specific to S and N

Antibody ELISAs were carried out as previously described (17), with 50 µl per dilution used. In brief, 96 well high-binding plates (Corning) were coated with 0.1 µg S or N protein in PBS and incubated overnight at 4°C. PBS-0.1% Tween 20 was used to wash plates 3 times, and between all subsequent steps. Plates were blocked with 2% (w/v) BSA in PBS-0.1% (v/v) Tween 20 for 1 hr at room temperature (RT). Serum was diluted 1:40 and incubated for 1 hr at RT. HRP-conjugated anti-human secondary antibodies were added for 1 hr at RT. For combined anti-IgG, IgA and IgM (IgGAM) the antibodies came in a combined pre-diluted form from The Binding Site (EACONJ654). The individual constituent HRP-labelled secondary antibodies used in this are polyclonal rabbit anti-human IgG (1:16,000), polyclonal rabbit anti-human IgA (1:2000) and polyclonal rabbit anti-human IgM (1:8000). Individual immunoglobulin isotypes were detected using HRP-conjugated monoclonal antibodies: mouse anti-human IgM (clone AF6, 1:2000), mouse anti-human IgG1 (clone MG6.41, 1:3000), mouse anti-human IgG3 (clone MG5.161, 1:1000). All monoclonal antibodies were produced at the University of Birmingham. Plates were developed for up to 20 minutes using 100 µl TMB Core (Bio-Rad) and the reaction was stopped with 50µl 0.2M H_2_SO_4_. Optical density (OD) was read at 450 nm using a SpectraMax ABS Plus plate reader.

### Solid Phase C1q-Binding Assay

Plates were coated as above. Plates were washed three times with PBS-0.1% Tween 20 – this wash step was carried out between all subsequent steps. Blocking was carried out for 1 hr at RT with 2% BSA in PBS-0.1% Tween 20. Test serum was heat inactivated at 56°C for 30 minutes, before being diluted 1 in 5 with 2% BSA supplemented with 5 mM calcium chloride and 5 mM magnesium chloride. 50 µl was added to the antigen-coated plate and incubated for 1hr at 37°C. After washing, 50 µl COVID negative normal human serum (same source used throughout all assays, containing no detectable S or N specific antibodies as measured by IgGAM ELISA) at a dilution of 1:40 (in 2% BSA plus 5 mM calcium chloride and 5 mM magnesium chloride) was added to each well for 1hr at RT. 100µl of rabbit anti-C1q FITC antibody (Invitrogen PA5-16601) at a 1:200 dilution in PBS-0.1% Tween 20 was added and incubated at 37°C for 1 hr. HRP conjugated swine anti-rabbit (Dako P0399) at a 1:2000 dilution was then incubated for 1 hr. The assay was amplified using the Perkin Elmer ELAST amplification kit as per manufacturer’s instructions, with an optimised dilution of streptavidin, 1:800, incubated for 20 minutes. Plates were developed using 100 µl TMB Core (Bio-Rad) for 10 minutes, before being stopped with 50 µl 0.2MH_2_SO_4_. OD was measured as described above.

### C4b, C3b and C5b Complement Deposition Assay

Microtiter plates were coated and washed as described above and blocked with Starting Block (ThermoFisher) for 10 min. Test serum was heat inactivated at 56°C for 30 minutes, before being diluted 1 in 5 with Starting Block supplemented with 5 mM calcium chloride and 5 mM magnesium chloride. 50 µl was added to the antigen-coated plate and incubated for 1hr at 37°C. After washing, 50 µl COVID negative normal human serum (same source used throughout all assays, containing no detectable S or N specific antibodies as measured by IgGAM ELISA) at a dilution of 1:40 (in 2% Starting Block plus 5 mM calcium chloride and 5 mM magnesium chloride) was added to each well for 1 hr at 37°C. The following anti-human monoclonal complement antibodies (100ul, diluted in PBS-0.1% Tween 20) were added and incubated at 37°C for 1 hr: mouse anti-C4b, 1:22,500 (Invitrogen, LF-MA0198); mouse anti-C3b, 1:10,000 (Invitrogen MA1-70053); mouse anti-C5b, 1:10,000 (Invitrogen DIA 011-01-02). HRP conjugated goat anti-mouse at a 1:4000 (Southern Biotech 1010-05) was then incubated at RT for 1 h. Plates were developed and read as described above.

### Statistics

Statistical analysis was carried out using GraphPad Prism 9.0. Kruskal-Wallis followed by Dunn’s *post-hoc* test for multiple groups was used to calculate p values. Statistical significance was accepted at P<0.05. Spearman correlation was carried out on the appropriate data sets.

## Results

### Anti-S, but Not Anti-N Antibody Responses Differ Between NHC and ITU-CONV Patients

**Figure 1:**
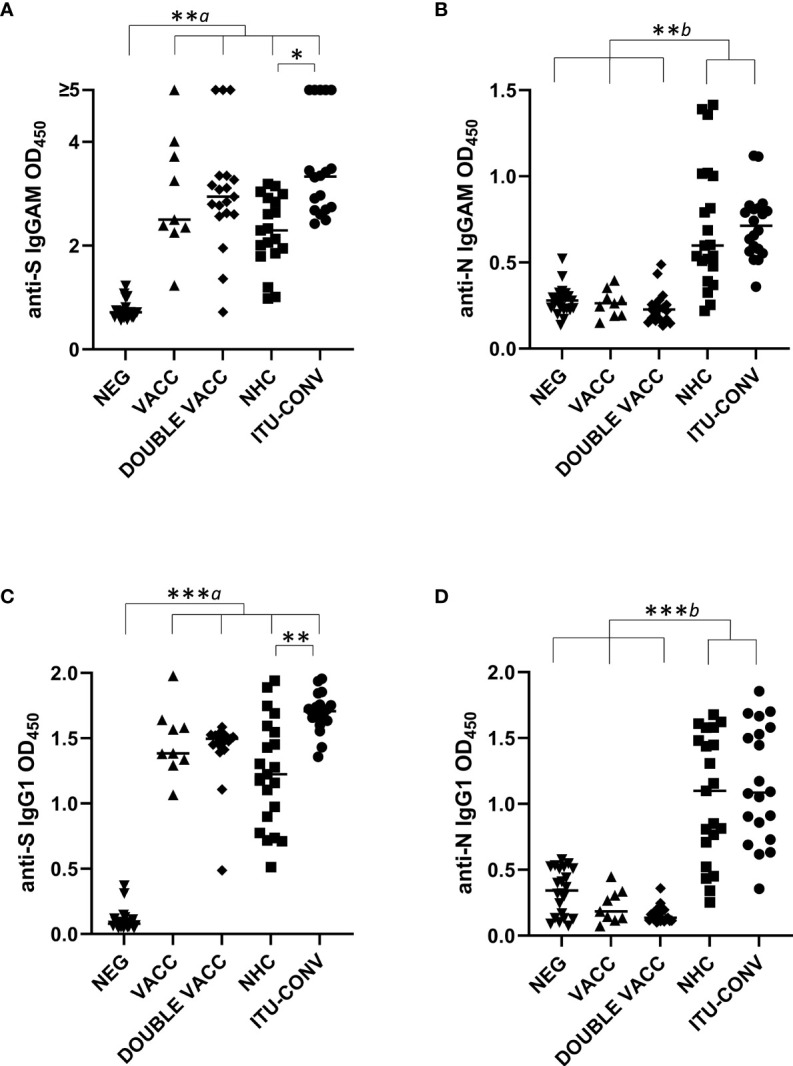
Anti-S, but not anti-N antibody responses differ between NHC and ITU-CONV patients. Using an ELISA against 0.1ug S **(A, C)** or N **(B, D)** with HRP-conjugated IgGAM or IgG1 secondary antibodies, GAM and IgG1 levels were assessed in the following subject groups: COVID-19 negatives (NEG, n ≥20), COVID-19 naïve one month post first BNT162b2 vaccine (VACC, n = 9), COVID-19 naïve one month post second BNT162b2 vaccine (DOUBLE VACC, n = 19), COVID-19 positive non-hospitalised convalescents (NHC, n ≥ 19) and COVID-19 positive convalescents who required ITU treatment (ITU-CONV, n ≥ 18) Kruskal-Wallis with Dunn’s multiple comparisons test was used to test significance. *a* indicates that the four groups bracketed (VACC, DOUBLE VACC, NHC and ITU-CONV) were individually significantly different to the NEG group; *b* indicates that NHC and ITU-CONV are independently significantly different to NEG, VACC and DOUBLE VACC. ***p < 0.001, **p < 0.01, *p < 0.05. Bars represent median values for each group.

Total IgGAM antibody responses to trimeric S and N were assessed in five different groups: individuals without any reported COVID-19 infection (NEG); post first BNT162b2 vaccine, infection-naïve individuals (VACC); post second BNT162b2 vaccine, infection-naïve individuals (DOUBLE VACC); convalescing non-hospitalised patients (NHC) and convalescing patients who had been hospitalised and required ITU treatment (ITU-CONV). The VACC, DOUBLE VACC, NHC, ITU-CONV groups all had significantly higher anti-S glycoprotein IgGAM responses than the NEG group (**Figure 1A**), whereas IgGAM levels against N in the two convalescent groups were higher than the NEG, VACC and DOUBLE VACC groups (**Figure 1B**). There were no significant differences in the anti-N responses between the NEG group and the VACC and DOUBLE VACC groups, consistent with individuals within these groups not having had prior SARS-CoV-2 infections. Similar results were observed when specific IgG1 responses, an IgG isotype efficient at fixing complement, were assessed (**Figure 1C**). No differences in anti-S IgGAM and IgG1 antibody responses were observed between the VACC and patient groups. Anti-S IgGAM and IgG1 responses were higher in the ITU-CONV group compared to the NHC group, but no differences were observed for anti-N responses in these two groups (**Figure 1D**). Modest IgM and IgG3 responses to S and N were detected in some individuals (**Supplementary Figures 1A, B** and **Supplementary c 1**).

### C1q Binding *in vitro* Correlates With Levels of S- and N-Specific IgGAM and IgG1 Antibodies

**Figure 2:**
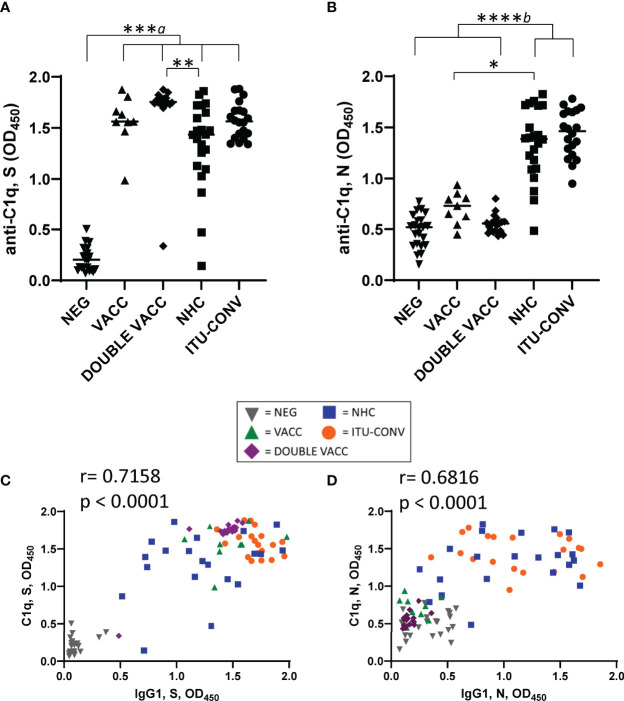
C1q binding to S and N correlates with IgG1 responses. Using an ELISA against 0.1ug S **(A)** or N **(B)** with an anti-C1q secondary antibody, followed by an HRP-conjugated tertiary, and the ELAST amplification kit, C1q binding was measured. Correlations of IgG1 OD and C1q OD against S **(C)** and N **(D)**. NEG, n = 22. VACC, n = 9. DOUBLE VACC n = 19. NHC, n ≥ 21. ITU-CONV n = 20. Kruskal-Wallis with Dunn’s multiple comparisons test was used. *a* indicates that the four groups bracketed were individually significantly different to the NEG group; *b* indicates that NHC and ITU-CONV are independently significantly different to NEG and DOUBLE VACC. ****p < 0.0001, ***p <0.001 **p < 0.01, *p <0.05. Bars represent median values for each group. Correlations were determined using the Spearman’s rank correlation test (r and p values presented).

To determine if the complement protein C1q can bind to SARS-CoV-2-specific immunoglobulins *in vitro* we developed a solid phase C1q-binding assay. In these antigen-specific assays, the test serum from COVID-19 patients or vaccinees is heat-inactivated and standardisation of complement is provided by using sera from non-infected, non-vaccinated subjects. Results from this assay showed that C1q binding mirrored IgG1 levels for both S and N antigens, with the lowest signals for S seen in the NEG group (**Figure 2A**), and for N in the NEG, VACC and DOUBLE VACC groups (**Figure 2B**). The lack of C1q binding detected for N when sera from the VACC and DOUBLE VACC groups were tested is expected and consistent with a lack of prior infection by SARS-CoV-2 in these groups (**Figure 2B**), and we have included these two groups in our downstream analyses of the response to N for their value as control groups. No difference in C1q binding was observed between sera from the two convalescent groups (**Figures 2A, B**). Plotting IgG1 responses against C1q responses shows a positive correlation between the amount of IgG1 antibody and the amount of C1q binding detected (**Figures 2C, D**). Therefore, C1q binding reflects the serological response to both antigens.

### Deposition of C4b, C3b and C5b Varies Dependent Upon Antigen Tested and Subject Group

**Figure 3:**
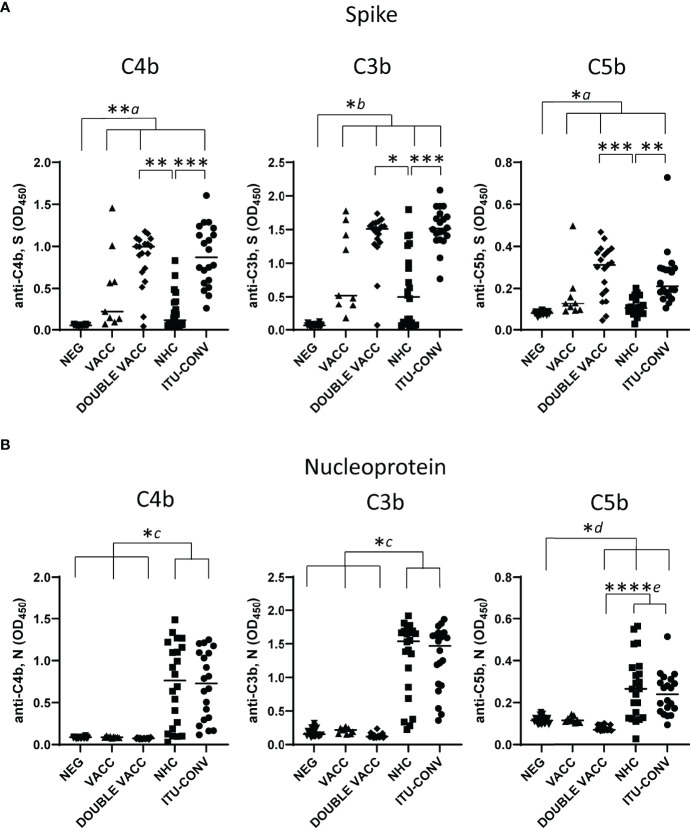
C4b, C3b and C5b show antigen and subject status-dependent variability. Using an ELISA against 0.1ug S **(A)** or N **(B)** with either anti-C4b, C3b or C5b secondary antibody, followed by an HRP-conjugated tertiary, downstream complement binding was measured. NEG, n = 22. VACC, n = 9. DOUBLE VACC, n = 19. NHC, n = 22. ITU-CONV n = 20. Kruskal-Wallis with Dunn’s multiple comparisons test was used. *a* and *b* indicate that the groups bracketed were individually significantly different to the NEG group; *c* indicates that NHC and ITU-CONV are independently significantly different to NEG, VACC and DOUBLE VACC; *d* indicates that DOUBLE VACC, NHC and ITU-CONV are all significantly different to NEG; *e* indicates that NHC and ITU-CONV are both significantly different to DOUBLE VACC. ****p < 0.0001, ***p < 0.001, **p < 0.01, *p < 0.05. Bars represent median values for each group.

To determine whether C1q binding reflected downstream activation of the complement cascade, we examined whether complement breakdown products could be detected. Deposition of C4b, a major component of the classical pathway C3 convertase, and the effector molecules C3b and C5b were assessed. In the absence of S or N-specific antibodies, C4b, C3b and C5b breakdown products were not detected, but they were detected in the presence of specific antibodies, indicating involvement of the classical complement pathway (**Figure 3**). When S was used as the assay antigen, the highest median levels of C4b, C3b and C5b deposition detected were in the DOUBLE VACC and ITU-CONV groups (**Figure 3A**), whereas the VACC and NHC groups showed similar lower levels of downstream activation. The median levels of C4b, C3b and C5b deposition detected when N was used as the assay antigen were similar between the NHC and ITU-CONV groups (**Figure 3B**). In contrast, for the NEG, VACC and DOUBLE VACC groups, where anti-N antibody responses and C1q binding were not detected, there was no downstream activation of the complement cascade. Therefore, in this assay differences in complement activation by antibody can be detected dependent upon what patient group and antigen were examined.

### Downstream Complement Activation Associates With Threshold IgG1 Responses

**Figure 4:**
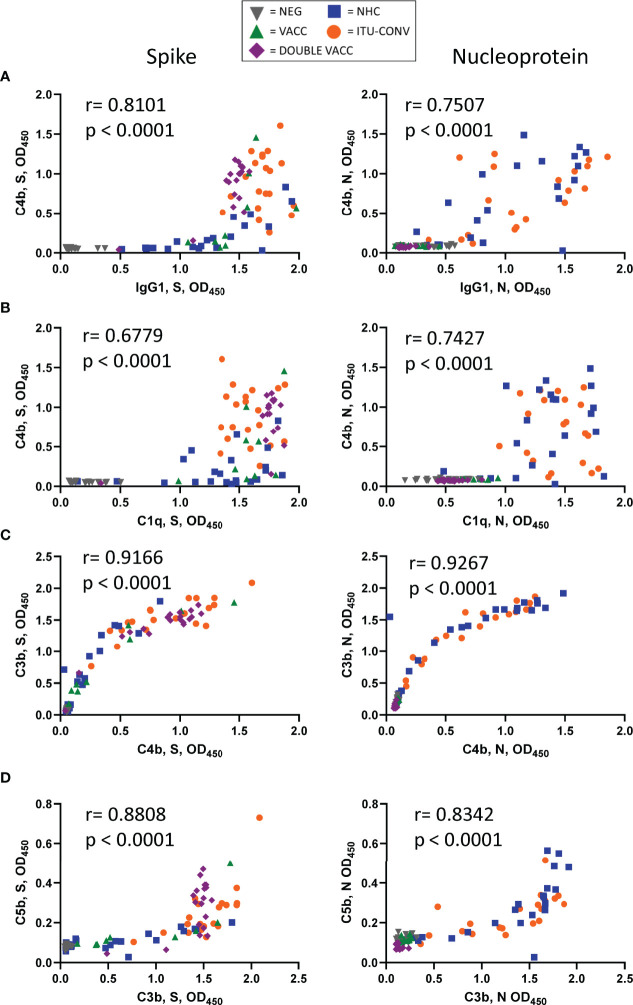
Threshold levels of IgG1 associate with the detection of C4b. **(A)** XY scatter plots for C4b responses and IgG1 responses, **(B)** C4b and C1q, **(C)** C3b and C4b and **(D)** C5b and C3b against S (left) or N (right). XY pairs, n ≥ 91, each point represents one serum. Correlations were determined using the Spearman's rank correlation test (r and p values presented).

In contrast to the linear association between anti-S IgG1 and C1q detection, there was a non-linear association between IgG1 and C4b, where C4b was only detectable beyond a threshold level of IgG1. A correlation between C4b and IgG1 to N was also observed, as was a threshold response, although the threshold response was less clear for N than for S (**Figure 4A**). Similar threshold responses for IgG1 to S and N were also observed if C3b or C5b were plotted against IgG1 (**Supplementary Figures 2A, B**). When correlations were performed for C1q vs C4b (**Figure 4B**), C4b vs C3b (**Figure 4C**) and C3b vs C5b (**Figure 4D**) for both antigens, then clear correlations were observed. This suggests that in this assay threshold levels of IgG1 are needed to activate downstream complement components for S and N.

### IgG1 Correlates With Complement Activation in Sera With the Lowest IgM Levels Detected

**Figure 5:**
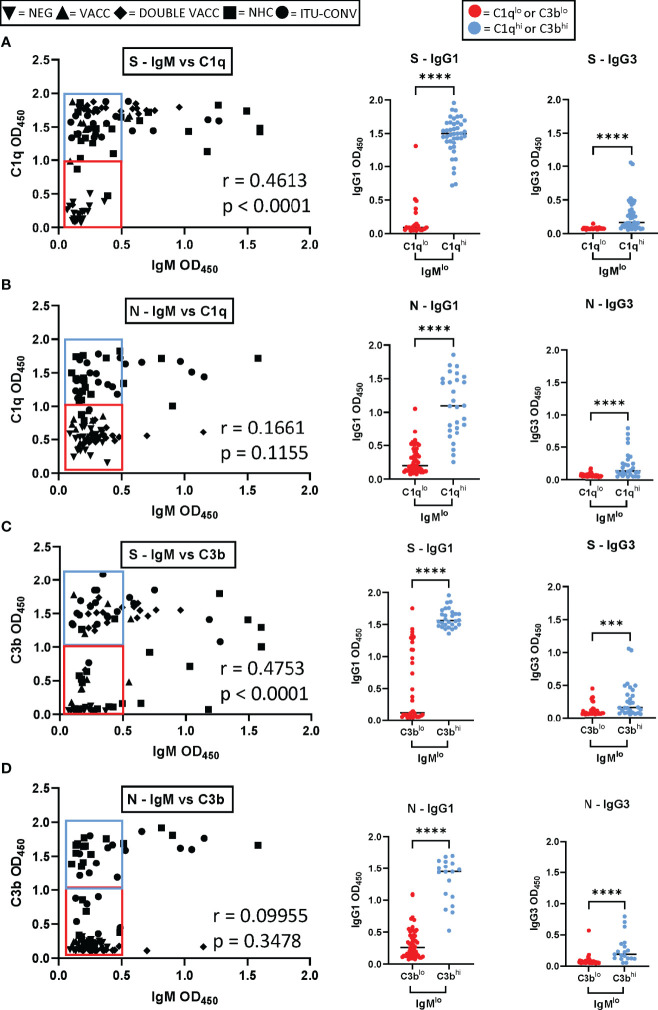
Complement activation by sera with low levels of IgM to S and N. **(A)** Left hand panel shows the IgM levels plotted against levels of C1q for S (left hand panel; results for 91 sera presented from NEG, VACC, DOUBLE VACC, NHC and ITU-CONV donors). The IgG1 levels for sera presented in the blue box (IgM^lo^C1q^hi^) or red box (IgM^lo^C1q^lo^) are shown in the central graph and for IgG3 in the right hand graph (n ≥ 18 for each group). Each coloured dot represents one sera from the corresponding coloured box. **(B)** As for a, but the anti-N response is presented. **(C, D)**, as for **(A, B)** respectively but with the results for C3b binding shown rather than C1q. Correlations were determined using the Spearman’s rank correlation test (r and p values presented). Mann-Whitney was used to test significance in the IgG1 and IgG3 column graphs, where ****p < 0.0001 and ***p < 0.001. Bars represent median values for each group.

IgM is the most efficient antibody isotype for fixing and activating complement (25) yet most sera tested had only low levels of anti-S or anti-N IgM (**Supplementary Figure 1A**). This was expected due to the convalescent nature of our infected groups, and the minimum one month period after vaccination that sera were obtained from our vaccinated groups. This suggested IgG1 or IgG3 might compensate for IgM when IgM is not present at high levels. Therefore, we examined the level of complement fixation (C1q binding) and activation (C3b deposition) associated with antigen-specific IgM and how this correlated with antigen-specific IgG1 and IgG3 levels (**Figure 5** and **Supplementary Figure 3**). To do this the IgM to S or N was correlated with C1q and C3b. There were modest correlations between IgM against S and C1q and C3b (r = 0.4613, p < 0.0001 and r = 0.4753, p < 0.0001 respectively; **Figures 5A, C**). To N protein, no correlation was identified, although only a few sera had elevated levels of IgM (IgM vs C1q, r = 0.1661, p = 0.1155 and IgM vs C3b r = 0.09955, p = 0.3478; **Figures 5B, D**) and if the 2 vaccinated groups (lacking anti-N responses) were excluded from the analysis for N, then the correlation became stronger (IgM vs C1q, r = 0.3019. p = 0.0162; IgM vs C3b, r = 0.03509, p = 0.0048). These results are consistent with IgM being able to activate complement when present, but not always being present at sufficiently high levels to do so. C1q binding and C3b deposition was detected for many sera in which IgM responses to S or N were low. We hypothesized that sera which exhibited the lowest IgM responses, but which could still fix and activate complement had higher levels of IgG1 or IgG3. We therefore divided the sera with the lower IgM levels into 2 groups, one group in which we detected the highest C1q or C3b levels (termed IgM^lo^C1q^hi^ and IgM^lo^C3b^hi^ respectively) and another group where we detected the lowest C1q and C3b levels (termed IgM^lo^C1q^lo^ and IgM^lo^C3b^lo^ respectively). The C1q or C3b response was then plotted against the IgG1 or IgG3 response. In the IgM^lo^C1q^hi^ and IgM^lo^C3b^hi^ groups higher levels of IgG1 were detected than in the IgM^lo^C1q^lo^ and IgM^lo^C3b^lo^ groups (**Figures 5A–D** and **Supplementary Figure 3**), and this difference was observed for both S and N. IgG3 levels were also higher in the IgM^lo^C1q^hi^ and IgM^lo^C3b^hi^ groups than in the IgM^lo^C1q^lo^ and IgM^lo^C3b^lo^ groups (**Figures 5A–D** and **Supplementary Figure 3**). Therefore, complement fixation and activation is observed in the presence of IgG1 or IgG3 when IgM levels are low.

## Discussion

Here we show (i) that antibodies to S and N can activate the classical complement cascade and (ii) the level of activation of the cascade detected vary dependent upon the subject group examined and the antigen used in the test. Therefore, antibodies to two different antigens within the same pathogen can activate the complement cascade *in vitro*, albeit at levels that depend on the severity of COVID-19. In the presence of specific antibodies to both S and N, similar levels of C1q binding were observed and the greatest variability was detected downstream of C1q binding. In contrast, in the NEG, VACC and DOUBLE VACC groups, where antibodies to N were not detected, activation of the complement cascade was not detected.

In this study, antibody needed to bind antigen in order to detect complement activation. Although threshold IgG1 levels were associated with activation of the complement cascade, the spread in the complement responses observed to spike and nucleoprotein did show some differences (e.g. **Figure 3**). Such observations may reflect the assay or other factors such as how antibodies themselves interact with these antigens or intrinsic differences in the antigens themselves. For instance, S is a trimeric protein and the approximately 420 kDa trimer is substantially larger than N, which is approximately 46 kDa. This difference may influence how IgG binds to the antigen and affects complement activation. Moreover, the trimeric structure of S means identical epitopes can be juxtaposed to one another in a highly defined manner (18, 26, 27) enabling multiple Fab to bind their target epitopes and promoting cross-linking of antigen. Other factors may also contribute to this process, such as which epitopes are targeted within the S protein. For instance, large-scale changes in the glycosylation pattern of S proteins has a surprisingly limited impact on the level of antibody binding by patient sera as determined by ELISA (19). One interpretation of this is that there are only a limited number of antibody sites available on each S glycoprotein for antibodies to bind, presumably in part because of a combination of relatively few (proteinaceous) epitopes targeted and the steric effects of antibodies themselves. This is similar to what we have observed for epitope recognition by antibodies targeting cell surface proteins on the surface of *Salmonella enterica* serovar Typhimurium (28). Moreover, studies examining antibody responses to the S glycoprotein of the Omicron variant in sera from vaccinated individuals suggest that amino acid changes in the relatively hypoglycosylated RBD (23, 29, 30) region can have dramatic effects on antibody binding (31) and so may be more likely to affect the level of complement activation. Other factors that may influence complement activation include how antigen is distributed on the surface of the virus (for S) and elsewhere in the host after natural infection. In the context of this assay, technical factors such as how antigen binds to the plate may influence the results observed. Furthermore, the trimeric vs monomeric nature of the antigens tested, the maintenance of native conformation, and the level of antigen denaturing may all influence the results observed using these assays. This highlights the need to develop multiple approaches to study antibody-antigen interactions *in vivo* to contextualise the *in vitro* results presented here.

IgM is the most efficient antibody isotype for activating the complement cascade (25). Nevertheless, as seen in many studies, the sera used in this study from individuals’ convalescent post-vaccination or infection, had modest or background levels of antigen-specific IgM detected. Those IgM^lo^ sera that could bind complement had higher IgG1/3 levels than those IgM^lo^ sera that could not activate the complement cascade. Therefore, the results from this study are possibly most relevant for understanding the relationship between IgG isotypes and complement activation, whereas the more pronounced complement-fixing properties of IgM may modulate the strengths of the responses observed. It is likely that in the presence of high levels of IgM, such as during the acute phase of a primary infection, there would be enhanced levels of complement activation. Nevertheless, the activation of the complement cascade in the absence of IgM may be possible and important for the pathophysiology of disease in other scenarios, as well as in the convalescent subjects we have presented here. For instance, many children with severe complications from COVID-19 (Multisystem Inflammatory Syndrome in Children (MIS-C)/paediatric inflammatory multisystem syndrome temporally associated with SARS-CoV-2 infection (PIMS-TS)) present with disease at times when they have low/negligible levels of IgM, yet high levels of IgG, especially IgG1 and IgG3 (32). This correlates with a strong signature associated with complement activation (33). Collectively, this likely means that IgM can play important roles in activating the complement cascade, but that it is not essential.

The results from these studies lead to further hypotheses to test. For instance, it is known that ITU subjects can have greater activation of the complement cascade (11, 12), and this could be compounding their disease. Indeed, targeting both C3 and C5 within the complement cascade as a way to treat COVID-19 shows promise (34–37). The availability of assays such as those described here will help increase understanding of how these inhibitors act to interfere with the complement cascade in the presence of antibody to different SARS-CoV-2 proteins. Alternatively, since all these subjects survived severe COVID-19 infections it could be hypothesised that the activation of complement is associated with a beneficial outcome. As we did not have sera from individuals who died this is not testable here. One caveat in this argument is that exogenous sources of complement in the form of sera from non-infected individuals were used in these studies and that patients own sera may differ in potency, or polymorphisms in complement components themselves could influence the consequences of complement activation (38, 39). This was not assessed here as the focus was on antibody-mediated activation of complement where we have attempted to standardize the amount of complement available, particularly for the downstream complement components. Additionally, these studies were performed using sera from patients who were infected or immunized weeks previously and the antibodies present may not reflect the antibodies present at the time of infection. Certainly, it could be expected that the affinity of the antibodies would increase over time. One striking feature was the variability in the anti-C4b/C3b response detected in the VACC group. It is unclear why this is the case, but it could simply be that there is variability within the wider population in the ability to activate complement downstream. Ultimately, the results generated by these assays are trying to recapitulate the complexity of the complement cascade *in vivo*, which is considerably more complex than what is observed or testable *in vitro*. For these reasons, it is important to interpret the outcomes from these assays as the starting point for further investigations. For instance, in these assays potential inhibition of activation is not attempted. Neither is the separation of competition for epitopes between antibody isotypes that are associated with greater complement-activating potential (e.g. IgM or IgG1) than others (e.g. IgA), or controlling for the absolute levels of each isotype present. Nevertheless, the differences observed may be indicative of differences that can occur in the patients in different tissues or organs or at different stages of infection.

The complement cascade has been reported to be activated through multiple pathways after SARS-CoV-2 infection (40–43). Amongst these, the engagement of the classical pathway is distinct to the non-antibody-dependent pathways due to the potential multiple roles antibody can play during the course of infection. If induced whilst an infection is ongoing, then the activation of the complement cascade by antibody could worsen disease, particularly as antibody responses become detectable concomitant with risk of severe disease. This could happen either through enhanced inflammation (41), such as observed during acute respiratory distress syndrome, or through enhancing the complications of thrombosis and coagulopathy after infection (44, 45). Moreover, antibodies are induced to multiple SARS-CoV-2 antigens and as we show, antibodies to S and N proteins have the capacity to activate complement *in vitro*. At the sites where complement is activated during primary infection, there could be additional increases in the anaphylatoxins C3a, C4a and C5a, as has been reported (11, 12, 15, 46). These could augment local inflammation through promoting the recruitment of more neutrophils and monocytes leading to tissue damage and worsen disease in patients. In addition, in humans challenged with influenza virus increased levels of C3a and C5a were detected in the upper respiratory tract (47), intriguingly most often during the recovery phase rather than the acute infection phase, presumably concomitant with when antibody responses are established. Other effects of complement activation may include the destruction of host cells due to the formation of the membrane attack complex (MAC) and the activation of the coagulation cascade and effects on the vasculature (38, 46, 48, 49).

Balancing this, positive roles for antibody-mediated complement activation have also been proposed during active infection and vaccination (14). Potentially, the most valuable contribution antibody-mediated complement activation could make to protection is in vaccinated individuals or in those recovered from prior infection. In such individuals, the level of antigen present is likely to be low when antibody encounters its target antigen. This binding of antigen by pre-existing antibody will still result in immune cell recruitment and activation, helping to prevent the wider dissemination of the virus, but the overall magnitude of these sequelae will be lower. This reduced level of inflammation compared to what is observed when antibody is induced during infection could result in reduced levels of immunopathology. Therefore, antibody-mediated activation of complement in this context may be more beneficial for the host because it is contributing to control of infection when the pathogen burden is relatively low and less likely to provoke severe inflammatory responses.

The site of pathogen encounter is likely to influence the type of antibody present, the amount of complement and the outcomes from complement activation. At mucosal surfaces there is an enrichment of anti-pathogen IgA as well as the presence of IgG and IgA is less efficient at activating complement than IgM, IgG1 and IgG3 (50, 51). In saliva, which is often used as a proxy for mucosal responses, both IgA and IgG to SARS-CoV-2 are readily detectable after infection (52, 53), but this is less so after vaccination alone (54). In contrast, IgM is typically not a major component of the antibody repertoire detected in saliva (55). Although there are significant levels of complement in the upper and lower respiratory tract (56, 57), the relative predominance of IgA may mean there is less activation of the complement cascade even between the upper and lower respiratory tracts. Limiting any inflammation or immunopathology associated with complement activation may be beneficial for maintaining barrier integrity. Nevertheless, significant levels of complement and its breakdown components are found in the respiratory tract after many different infections (58–60). This means that complement can be activated in the respiratory tract and so in some circumstances may have negative impacts on the host.

In summary, we have identified activation of the classical complement pathway after vaccination against COVID-19, or after COVID-19 infection. Future studies will help us further understand how complement is activated in the presence of antibodies and how this may contribute to protection and harm in those who encounter this pathogen.

## Data Availability Statement

The original contributions presented in the study are included in the article/**Supplementary Material**. Further inquiries can be directed to the corresponding author.

## Ethics Statement

The studies involving human participants were reviewed and approved by London – Camden and Kings Cross Research Ethics Committee reference 20/HRA/1817 and North West ethics committee, Preston CIA UPH IRAS approval reference REC 20\NW\0240. The patients/participants provided their written informed consent to participate in this study.

## Author Contributions

Conceptualization: EM-J, AS, LH, IH, JR, DW, MC, MD, AR, AC. Methodology: RL, EM-J, MP-T, SJ. Investigation: RL. Data analysis: RL. Resources: SF, MG, MN, IC, TD, TV, AS, AR. Supervision: SW, AC. Writing – original draft: RL, AC. All authors reviewed and edited the final manuscript.

## Funding

This work was supported by the Wellcome Trust Mechanisms of Inflammatory Disease (MIDAS) PhD Programme [grant number 222389/Z/21/Z, part of 108871/B/15/Z] to RL; The Royal Society Newton International Fellowship [grant number NIF\R1\192061] to E.M.J and AC; a British Heart Foundation Intermediate Fellowship [grant number FS/IBSRF/20/25039] to JR; The University of Southampton Coronavirus Response Fund to MC and Medical Research Council [grant number MR/W010011/1] to LH, AR and AC.

## Conflict of Interest

The authors declare that the research was conducted in the absence of any commercial or financial relationships that could be construed as a potential conflict of interest.

## Publisher’s Note

All claims expressed in this article are solely those of the authors and do not necessarily represent those of their affiliated organizations, or those of the publisher, the editors and the reviewers. Any product that may be evaluated in this article, or claim that may be made by its manufacturer, is not guaranteed or endorsed by the publisher.
