# Supplementary material for: SARS-CoV-2 Spike- and Nucleoprotein-Specific Antibodies Induced After Vaccination or Infection Promote Classical Complement Activation
Source: Front Immunol. 2022 Jul 4;13:838780. doi: 10.3389/fimmu.2022.838780 (PMC9289266; doi:10.3389/fimmu.2022.838780)
Supplement: Supplementary file 1 [file DataSheet_1.docx]

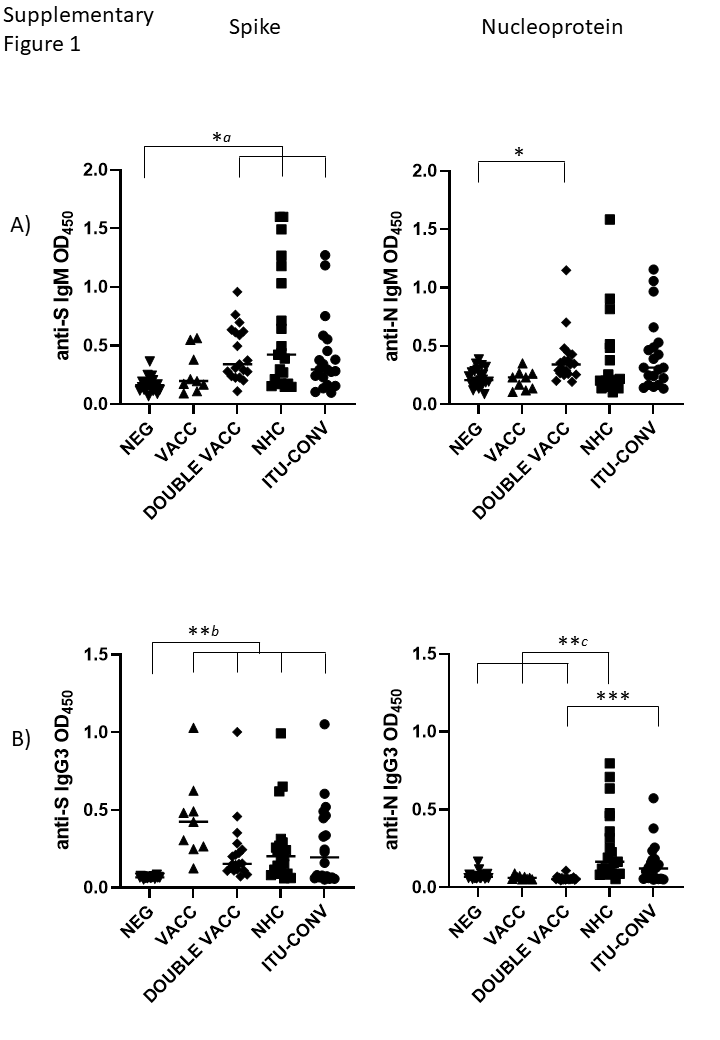
Supplementary Material

**Supplementary figure 1: Anti-S and N IgM and IgG3 responses in sera from different groups after infection or vaccination.** Serological responses were assessed by ELISA using HRP labelled a) anti-IgM and b) IgG3 against 0.1ug purified S or N. Kruskal-Wallis with Dunn’s multiple comparisons test was used to test significance. *a* and *b* indicate that the groups bracketed were individually significantly different to the NEG group; *c* indicates that NEG, VACC and DOUBLE VACC are independently significantly different to NHC. *** p < 0.001, ** p < 0.01, p < 0.05. Bars represent median values for each group. NEG, n = 22. VACC, n = 9. DOUBLE VACC, n = 19. NHC, n = 21. ITU-CONV, n = 20.


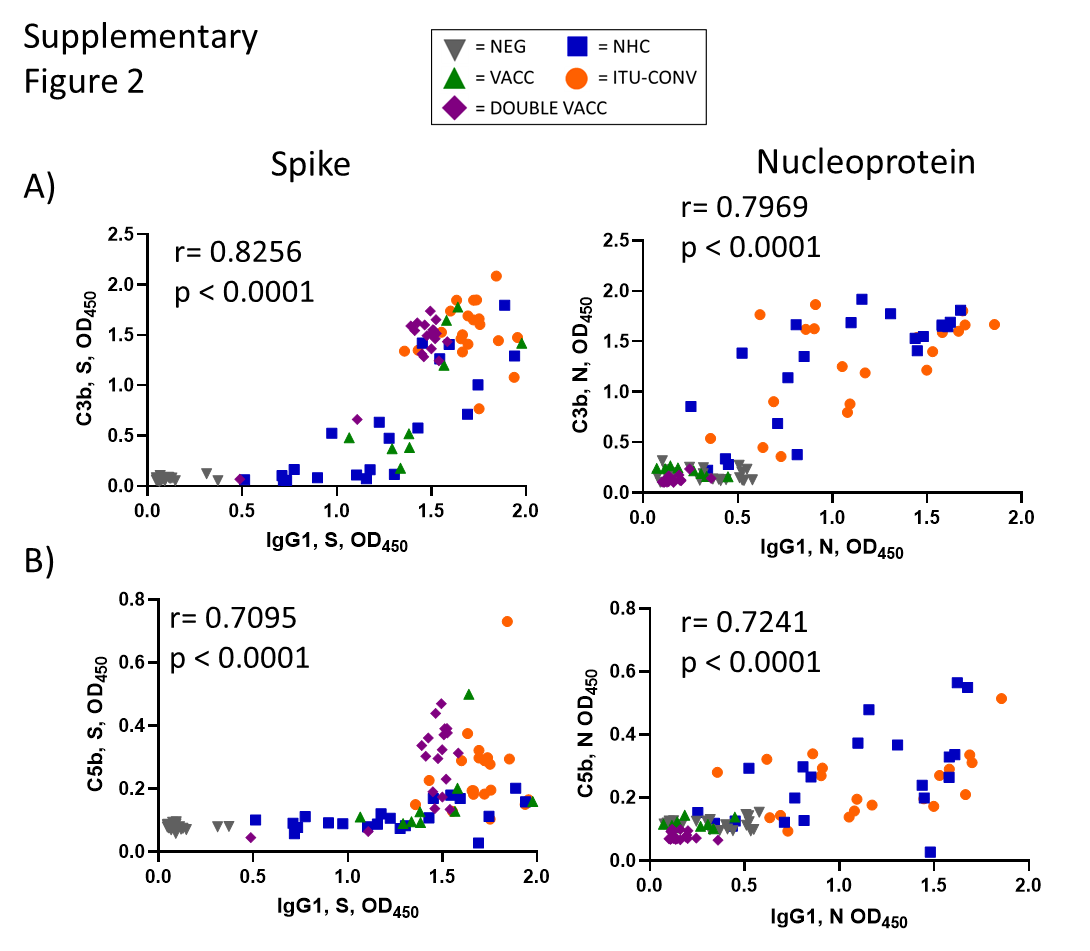


**Supplementary figure 2: Correlations between (A) IgG1 and C3b or (B) IgG1 and C5b for S and N.** Correlations of data obtained in Figs 1c and 1d, and Figs 3a and 3b were plotted. XY pairs, n = 91. Spearman correlation was used to assign r and p values.

**
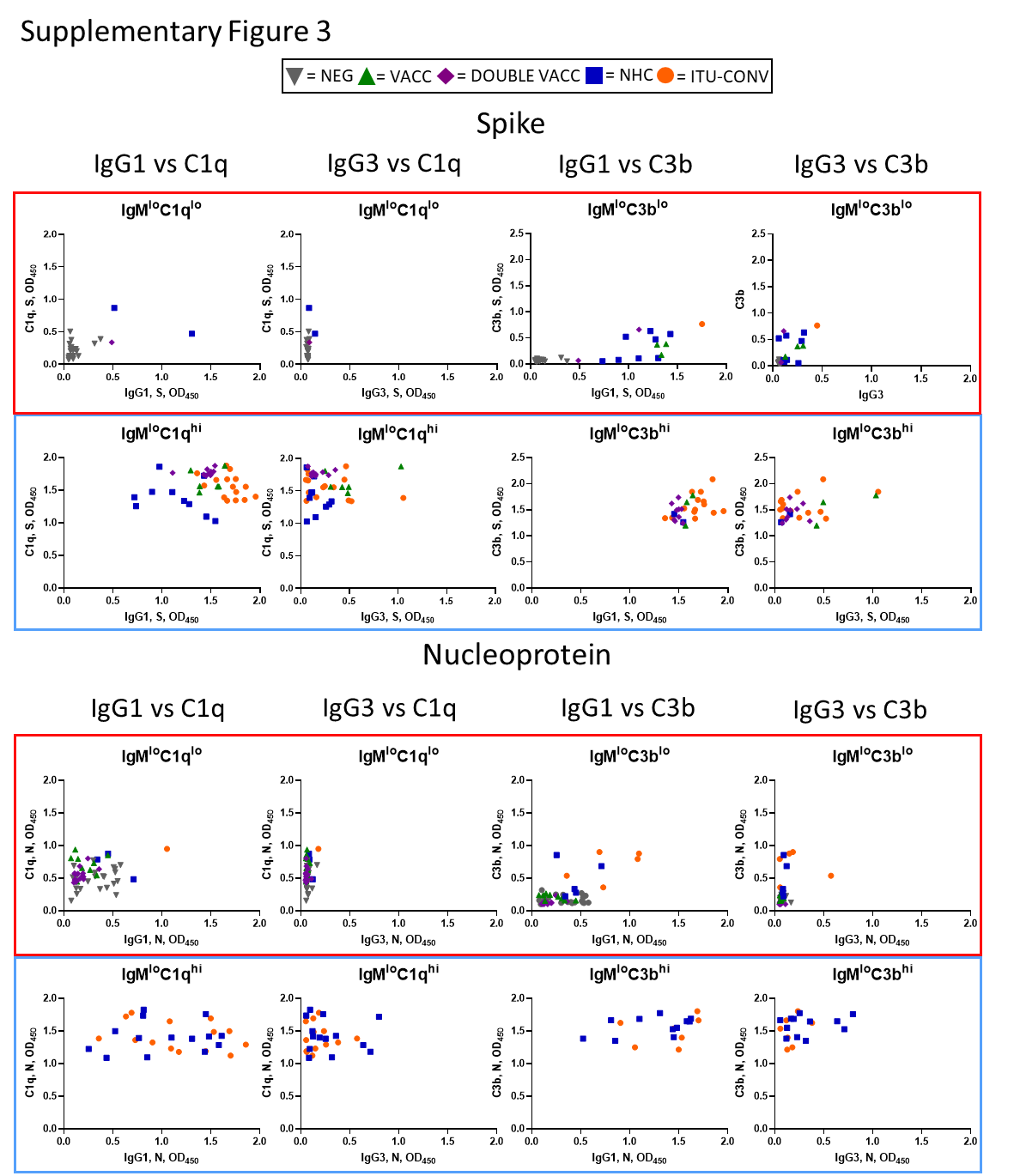
**

**Supplementary Figure 3. Results of comparisons between IgG1 or IgG3 and C1q and C3b from sera with low levels of IgM to S and N.** Scattergraphs showing IgG1 vs C1q, IgG3 vs C1q, IgG1 vs C3b or IgG3 vs C3b for the sera contained in the blue or red boxes shown in Figure 5.

Supplementary Table 1

|  | NEG | VACC | DOUBLE VACC | NHC | ITU-CONV |
| --- | --- | --- | --- | --- | --- |
| S IgGAM | 0.72 | 2.50 | 2.95 | 2.28 | 3.32 |
| N IgGAM | 0.28 | 0.26 | 0.22 | 0.60 | 0.72 |
| S IgG1 | 0.07 | 1.38 | 1.49 | 1.22 | 1.71 |
| N IgG1 | 0.34 | 0.18 | 0.14 | 1.10 | 1.09 |
| S IgM | 0.16 | 0.20 | 0.33 | 0.42 | 0.30 |
| N IgM | 0.20 | 0.23 | 0.34 | 0.20 | 0.31 |
| S IgG3 | 0.07 | 0.42 | 0.15 | 0.20 | 0.19 |
| N IgG3 | 0.07 | 0.06 | 0.05 | 0.16 | 0.12 |

**Supplementary Table 1: Summary table of median antibody optical density values against S and N in different patient groups.** Median values obtained from data in Figure 1 and Supplementary Figure 1. n ≥ 18 for all groups.
